# The Effect of Complete Integration of HIV and TB Services on Time to Initiation of Antiretroviral Therapy: A Before-After Study

**DOI:** 10.1371/journal.pone.0046988

**Published:** 2012-10-05

**Authors:** Bernhard Kerschberger, Katherine Hilderbrand, Andrew M. Boulle, David Coetzee, Eric Goemaere, Virginia De Azevedo, Gilles Van Cutsem

**Affiliations:** 1 Médecins sans Frontières, Khayelitsha, Cape Town, South Africa; 2 Centre for Infectious Disease Epidemiology and Research, School of Public Health and Family Medicine, Faculty of Health Sciences, University of Cape Town, Cape Town, South Africa; 3 South African Medical Unit, Médecins sans Frontières, Johannesburg, South Africa; 4 City of Cape Town, Health Directorate, Khayelitsha, South Africa; Vanderbilt University, United States of America

## Abstract

**Background:**

Studies have shown that early ART initiation in TB/HIV co-infected patients lowers mortality. One way to implement earlier ART commencement could be through integration of TB and HIV services, a more efficient model of care than separate, vertical programs. We present a model of full TB/HIV integration and estimate its effect on time to initiation of ART.

**Methodology/Principal Findings:**

We retrospectively reviewed TB registers and clinical notes of 209 TB/HIV co-infected adults with a CD4 count <250 cells/µl and registered for TB treatment at one primary care clinic in a South African township between June 2008 and May 2009. Using Kaplan-Meier and Cox proportional hazard analysis, we compared time between initiation of TB treatment and ART for the periods before and after full, “one-stop shop” integration of TB and HIV services (in December 2009). Potential confounders were determined a priori through directed acyclic graphs. Robustness of assumptions was investigated by sensitivity analyses. The analysis included 188 patients (100 pre- and 88 post-integration), yielding 56 person-years of observation. Baseline characteristics of the two groups were similar. Median time to ART initiation decreased from 147 days (95% confidence interval [CI] 85–188) before integration of services to 75 days (95% CI 52–119) post-integration. In adjusted analyses, patients attending the clinic post-integration were 1.60 times (95% CI 1.11–2.29) more likely to have started ART relative to the pre-integration period. Sensitivity analyses supported these findings.

**Conclusions/Significance:**

Full TB/HIV care integration is feasible and led to a 60% increased chance of co-infected patients starting ART, while reducing time to ART initiation by an average of 72 days. Although these estimates should be confirmed through larger studies, they suggest that scale-up of full TB/HIV service integration in high TB/HIV prevalence settings may shorten time to ART initiation, which might reduce excess mortality and morbidity.

## Introduction

In sub-Saharan Africa, an estimated 22.5 million people were living with HIV/AIDS [1] and about 2.8 million [2] were diagnosed with TB in 2009. South Africa is especially hard-hit by the closely entwined TB and HIV epidemics, with an estimated adult HIV prevalence of 17.8% [1] and an annual TB incidence of 970/100,000 [2]. In 2009 about 490,000 new TB cases were diagnosed, of whom 58% were co-infected with HIV [2]. The two diseases form a vicious spiral: TB is the main cause of death among people living with HIV and AIDS [1], and HIV - which drives the TB epidemic - is one of the main reasons for South Africa’s failure to achieve TB control [2], [3]. In addition, an epidemic of multi-drug resistant tuberculosis (MDR-TB) has emerged from the intersection of these two diseases [4], [5].

HIV changes the clinical presentation of TB from a slowly progressing disease with reasonable prognosis to one with a high mortality rate [6]. TB-related mortality in HIV co-infected patients is particularly high during the first months of TB treatment [7]. Intervening more effectively during this critical period is therefore essential to saving lives.

Evidence from randomised clinical trials as well as observational data shows that early initiation of ART (between 2 and 8 weeks after the start of TB treatment) improves survival [8]–[15]. Based on these findings, WHO [16] and South African [17] guidelines therefore recommend ART initiation during this time period. However, in practice ART initiation is sometimes delayed [18] due to factors such as patient characteristics, overlapping drug toxicities, fear of clinicians, and national health policy.

Structural barriers at different levels within the health system also impede coordination of HIV/AIDS and TB activities, negatively impacting TB/HIV prevention and care [19]–[21]. For instance, a study from South Africa showed that ART initiation in TB clinics can be delayed as much as 116 days, mainly due to prolonged referral times in moving between TB and ART services [22]. Well-documented shortages in human resources for health care [23]–[26] also contribute to delays, and to primary health care services becoming overwhelmed by increasing numbers of people presenting with both diseases.

Clearly, continued implementation of vertical HIV/AIDS and TB programs that treat each disease separately is inadequate and must be replaced by new models of care. Vertical programs involve duplication of services, which leads to inefficient use of already-limited resources. In contrast, an integrated public health approach to disease control utilizes available resources more efficiently [27]. Varying levels of TB/HIV service integration have been discussed extensively [21], [28]–[30] and integration has shown to be feasible and sustainable in various settings in sub-Saharan Africa with dual TB and HIV epidemics [31]–[36]. However, models of integration are not clearly outlined, and different definitions of integration exist. A recent systematic review [21] of TB/HIV services in low and middle income countries described five models of integration, ranging from simple referral, to collaboration between HIV and TB services, and to complete integration of services. This review also highlighted the lack of formal evidence on the impact of TB/HIV service integration, and of comparative studies of outcomes under different models.

Since 2004 Médecins Sans Frontières (MSF), the Provincial Government of the Western Cape and the City of Cape Town have piloted several models of TB/HIV integration in Khayelitsha [20]. We hypothesized that complete service integration would lead to faster initiation of ART in TB/HIV co-infected patients. Our primary objectives were 1) to compare the time from the start of TB treatment to ART initiation in eligible TB/HIV co-infected adults before and after complete integration of TB and HIV services in a primary care clinic in a South African township; and 2) to describe a “one stop shop” model of fully integrated TB and HIV services. The broader aim is to develop an evidence base that informs public health policy on the impact of this model of care.

## Methods

### Study setting

**Figure 1:**
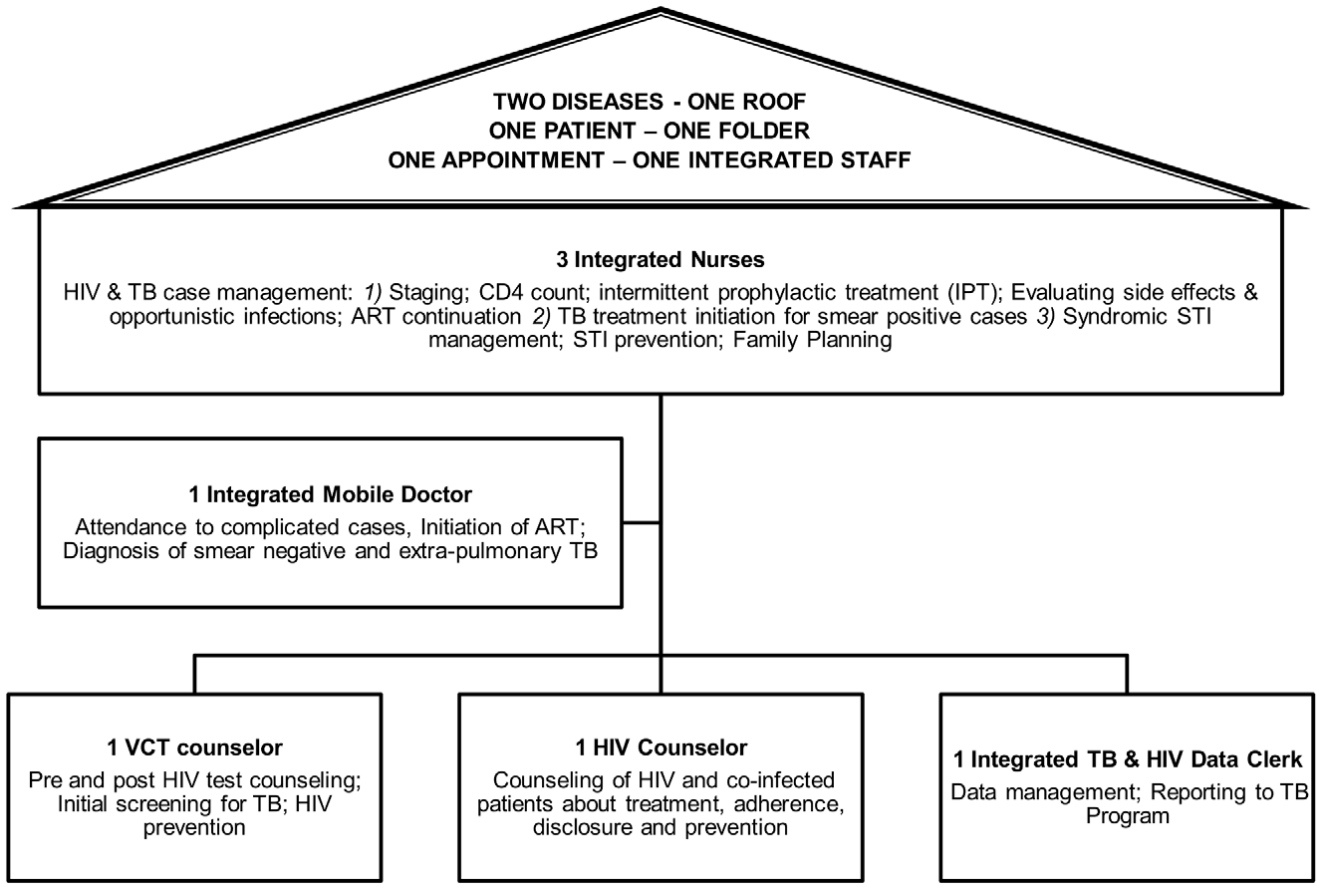
Roles of health staff in Town 2 clinic after TB/HIV service integration. Model of care as it was implemented in Town 2 clinic in December 2008.

The township of Khayelitsha in Cape Town has more than 500,000 inhabitants and among the highest HIV and TB infection rates both nationally and globally [37]. Town 2 clinic is one of 10 primary care clinics providing TB treatment, and treated 612 patients in 2008, out of an annual caseload of approximately 6000 TB cases for Khayelitsha. Until November 2008, Town 2 clinic provided vertical TB care, HIV testing, CD4 counts, cotrimoxazole prophylaxis and family planning, but co-infected patients eligible for ART were referred to other ART sites in the sub-district [37]. As of December 2008, ART initiation, pre- and post- ART follow-up were integrated into TB services. This resulted in a “one stop shop” TB/HIV model of care where both services are delivered by the same health professional at a single entry point (**Figure 1**). Services provided pre- and post-integration are described in **Table S1**.

#### Description of full ‘one stop shop’ TB/HIV integration

Service integration was implemented at three tiers of the health facility level: (1) administrative and managerial: one facility manager oversees the provision of all HIV and TB service activities; (2) health care provision: the same doctor, nurse, counsellor, pharmacist assistant and clerk attends to all patients, regardless of whether they have TB and/or HIV; and (3) monitoring and evaluation: a single health information system records data for both diseases in one patient file.

Infection control precautions are enforced through an infection control policy addressing administrative, environmental and individual protections. A poster describing the infection control policy in detail is displayed in every clinic (**Poster S1**). An Infection Control Committee, which consists of the facility manager, a health and safety representative, a cough officer, the TB/HIV nurses and a representative from the community, is responsible for enforcing the policy. Major administrative control items include six monthly infection control risk assessments; screening of health care workers; education sessions for health workers, patients and the community; cough triage; rapid diagnosis and initiation of TB treatment; contact tracing; display of TB signage and literature; and enforcement of environmental and personal controls. Environmental controls focus on maximising low maintenance natural ventilation and ensure appropriate directional flow in consultation rooms. Small infrastructural changes were implemented to ensure a minimum of 12 air changes per hour in every room; these include the installation of additional windows, wind-driven roof turbines, an outside sputum booth, and dry-wall partitioning to modify patient-flow. Personal respiratory protection consists of distribution of surgical masks to all coughing patients and N95 respirators to health care workers. In addition to facility-level infection control interventions, the MSF project team did regular education on infection control measures on local radio, and conducted a campaign entitled ‘Stop TB. Open your window’, targeting mini-van taxis and areas of congregation.

Training in HIV management and provision of ART was provided to all TB staff, and the clinic received reference tools (HIV/TB treatment and counselling guidelines). For a six month period, one additional nurse-mentor, one counsellor-mentor and one data clerk-mentor were added to the existing staff (3 nurses, one mobile doctor, one TB data clerk and one person for HIV counselling and testing [HCT]). The aim was to develop the skills of existing staff, provide support to manage the added HIV- and ART- related activities, and absorb the increased workload.

The resulting services offered integrated TB and HIV case management (including pre-ART care, ART initiation and follow-up), management of sexually transmitted diseases, HCT, family planning, and prevention. The integrated clinic enrolled patients with HIV and/or TB, including patients who were HIV infected but did not have TB, patients with TB who were HIV uninfected, and TB/HIV co-infected patients. Once a week the mobile doctor attended complicated medical cases and initiated ART in eligible patients. TB diagnosis was based on sputum specimens, smear microscopy or cultures, and/or the doctor’s clinical judgment [38]. HIV testing was offered to all TB patients. HIV-infected patients were systematically screened for TB at every consultation using the WHO symptom screen, enquiring on cough for more than 2 weeks, night sweats, loss of weight and fever. HIV co-infected patients were assessed for eligibility for cotrimoxazole preventive therapy, and CD4 cell count testing was performed. ART eligible patients attended three pre-ART counselling sessions. TB clients underwent directly observed treatment for the first 2 weeks. After this initiation, stable and adherent co-infected patients collected their antiretroviral drugs monthly and their TB drugs at weekly or monthly intervals (depending on individual needs) - all from the same pharmacy. Each patient had one medical file containing all TB and HIV notes, counselling sheets, screening tools, and prescription charts. Medical follow-up data were routinely entered into paper registers for ART and TB. To ensure complete TB cohort reporting, information on HIV and ART status, CD4 count and cotrimoxazole prophylaxis were also recorded in the TB register at the beginning of TB treatment.

### Study Design, Inclusion and Exclusion Criteria, and Definitions

This is a retrospective observational before-and-after study. To select patients for inclusion in the analysis, we reviewed TB registers and clinical files of TB/HIV co-infected patients aged >16 years who were eligible for ART and registered for TB treatment between June 1, 2008 and May 31, 2009 at the intervention clinic. A TB case was defined as anyone registered for TB treatment at Town 2 clinic, regardless of whether treatment was initiated at the study clinic or elsewhere. An HIV case was defined as anyone with an HIV positive test result recorded either before or within a month of starting of TB treatment. If two or more HIV test results were recorded for a given patient, the one closest to the TB treatment initiation date was considered. Patients on ART at the time of TB treatment initiation were excluded from the analysis. Those who received at least one dose of ART after starting TB treatment were classified as initiated on ART. Eligibility for ART was defined as anyone with a CD4 cell count at or below 250 cells/µl, measured 6 months before or after the TB treatment start date. South African national guidelines during this study’s period of observation recommended initiating ART in patients with WHO stage IV and/or CD4<200 cells/µl. However, we followed local clinical practice, which was to initiate ART at CD4<250 cells/µl. The South African TB guidelines at the time recommended initiation of ART after 2 months of TB treatment for patients with CD4 counts 50–200 cells/µL and/or WHO stage 4, and after 2 weeks for patients with CD4<50 cells/µL. Patients with CD4>200 cells/µL were not eligible for ART [38], [39].

### Data Management and Statistical Analysis

Data were recorded in an electronic database and checked for errors and inconsistencies. TB and HIV information were obtained from TB registers; identification information was matched either with an electronic HIV patient database or with paper-based ART registers from HIV referral clinics in Khayelitsha. Patient files were traced to obtain detailed HIV information and to validate records from TB registers. Each patient was followed until the date of an endpoint event, which was (1) ART initiation; (2) death; (3) last visit before being lost to follow up (LTFU), transferred out (TFO) or experiencing TB treatment failure; or (4) end of the study observation period (275 days after TB treatment start) for patients not initiated on ART. Patients known to have initiated ART during TB treatment but without recorded dates were assumed to have begun at the end of TB treatment. Those with unknown ART status were censored at the end of TB treatment.

We described and compared baseline characteristics of patients before and after service integration using Chi square or Fisher’s exact tests for categorical variables and Student’s t- or Wilcoxon rank-sum test for continuous variables. Estimates of median time to ART initiation were determined using Kaplan-Meier survival analysis. We used log-rank tests and Cox proportional hazards regression analysis to describe univariate associations between baseline factors and time to ART initiation. Potential confounders were determined a priori using directed acyclic graphs (DAGs, described in **Figure S1**), and were included in a multivariate proportional hazards Cox regression model. Variables were tested for interactions, and Schoenfeld residuals were used to validate the proportional hazard assumption. Collinearity was assessed by calculating the variance inflation factors (VIF). All data were analyzed using STATA version 11.0 (StataCorp, College Station, Texas, USA).

### Sensitivity Analysis

We also performed other sensitivity analyses (SA). Though not identified in DAGs as potential confounders, we included the variables TB classification and TB patient category (SA 1), excluded all patients who initiated TB treatment in another facility (SA 2), and categorized continuous variables (SA 5). Patients with unknown exact ART initiation dates during TB treatment were excluded (SA 3), or ART initiation was assumed to have occurred in the middle of TB treatment (SA 4). Finally, in accordance with national guidelines, only patients with CD4 cell counts equal to or less than 200 cells/µl were considered eligible for ART (SA 6).

### Ethics

The research protocol was approved by the Health Sciences Faculty Research Ethics Committee of the University of Cape Town (REC REF 222/2010) and the City of Cape Town Health Directorate Research Committee. Individual patient consent was waived by the Ethics Committee, consistent with the South African Medical Research Council’s Guidelines on Ethics for Medical Research and the Declaration of Helsinki, because this was a retrospective analysis of routine clinical service records, no additional data collection or procedures were undertaken from or on patients, all patient information was entered into the database using coded identification numbers, and no information that could reveal patient identity was entered into the database.

## Results

### Study Subjects and Baseline Characteristics

**Figure 2:**
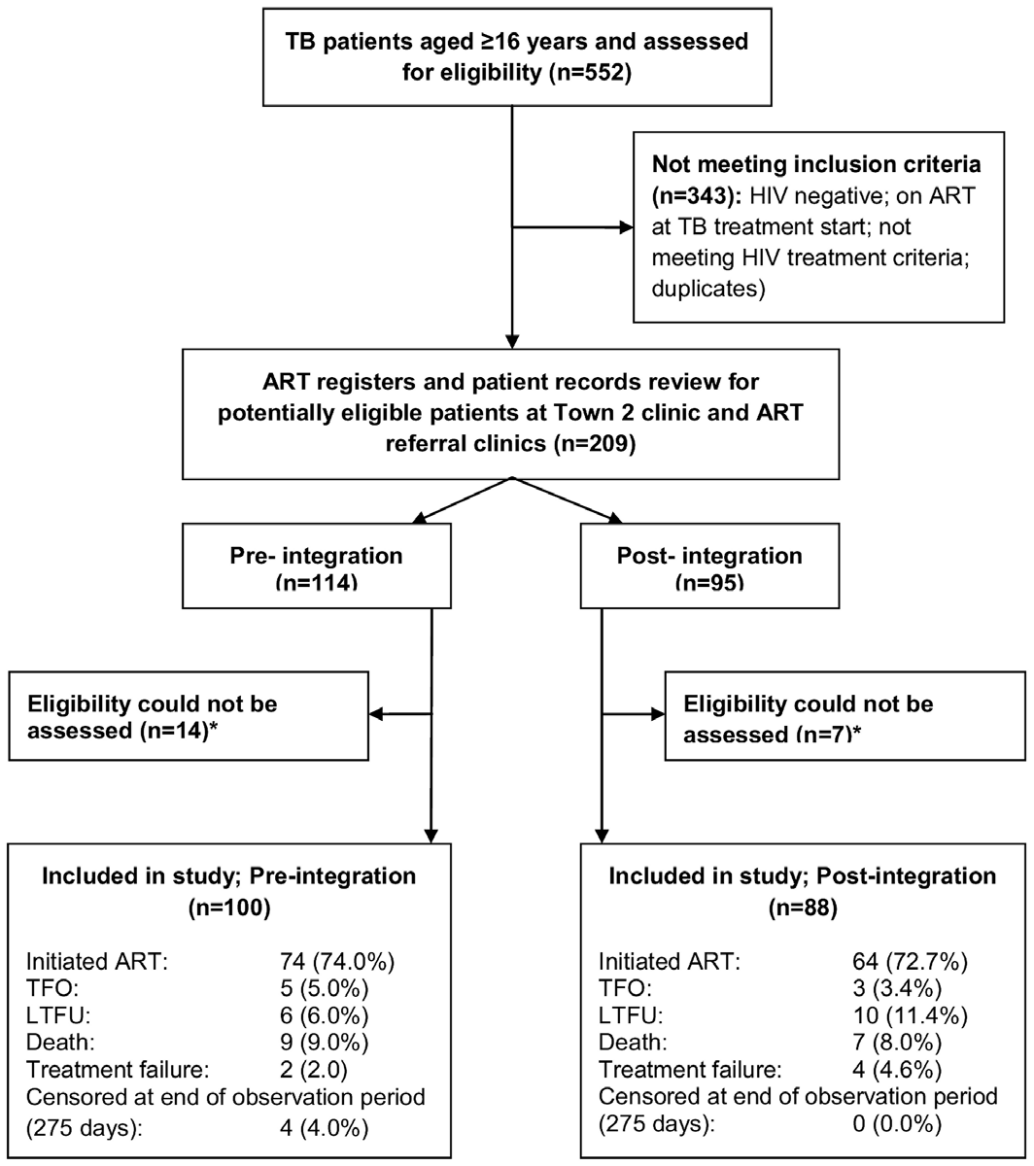
Flow chart of patients included in the study in Town 2 clinic from June 2008 to May 2009. Legend: TB, tuberculosis; ART, antiretroviral treatment; TFO, transferred out; LTFU, lost to follow-up.

We identified 552 TB patient records during the study period, of which 343 did not meet the inclusion criteria (**Figure 2**). Of the remaining 209 potential study participants, 21 (10.0%) were excluded because eligibility could not be assessed. There were no statistically significant differences in sex, age, TB patient category, TB classification or TB initiation (in referral clinic) between included and excluded participants (data not shown). In total, 343 TB patients (62.1%) were co-infected with HIV; 134 (39.1%) had CD4 cell counts >250 cells/µl and/or were on ART at the start of TB treatment.

The remaining 188 patients (90.0%) accrued 56 person-years of observation, with a median follow-up duration of 102.5 days (IQR 49.5–188) and 57 days (IQR 32–163.5) for the periods before and after service integration. Exact dates of ART initiation during TB treatment and ART status at the end of TB treatment were unknown for 15 and 16 patients respectively. The median age and CD4 cell count were 35 years (IQR 29–40) and 87 cells/µl (IQR 37–136). Half the patients were male; 73.1% (n = 136) were new TB cases; 38.8% (n = 73) commenced TB treatment in another clinic; and 64.4% (n = 121) and 30.3% (n = 57) were diagnosed with pulmonary or extra-pulmonary TB (**Table 1**). There were no statistically significant differences between patients before and after TB/HIV service integration regarding sex, age, CD4 cell count, TB patient category and TB initiation (**Table 1**). Seventy four (74.0%) pre-integration patients and 64 (72.7%) post-integration patients initiated ART during the observation period, and the remaining ones (death, LTFU, TFO, TB treatment failure, reaching end of observation period) were censored (**Figure 2**). Extra-pulmonary TB increased from 23.0% to 38.6% after integration, while pulmonary TB decreased from 70.0% to 58.0% (p = 0.051).

**Table 1:** Baseline characteristics of TB/HIV co-infected patients included in the study in Town 2 clinic from June 2008 to May 2009.

			Service integration		
		All	Before	After	p-value
Total enrolled		188	100	88	
Sex; n (%)	**Female**	95 (50.5)	50 (50.0)	45 (51.1)	0.876
	**Male**	93 (49.5)	50 (50.0)	43 (48.9)	
Age (years), median (IQR)		35 (29–40)	35.5 (28–41.5)	34 (30–40)	0.820
CD4 count; (cells/µl), median (IQR)		87 (37–163)	87.5 (50–169)	83.5 (31.5–157.5)	0.381
TB treatment category; n (%)	**New**	136 (73.1)	72 (72.7)	64 (73.6)	0.898
	**Re-treatment**	50 (26.9)	27 (27.3)	23 (26.4)	
TB initiation*; n (%). In clinic		115 (61.2)	59 (59.0)	56 (63.6)	0.775
Outside clinic	**1–14 days**	41 (21.8)	22 (22.0)	19 (21.6)	
	**15–60 days**	25 (13.3)	14 (14.0)	11 (12.5)	
	**≥61 days**	7 (3.7)	5 (5.0)	2 (2.3)	
TB classification; n (%)	**Pulmonary**	121 (64.4)	70 (70.0)	51 (58.0)	0.051
	**Extrapulmonary**	57 (30.3)	23 (23.0)	34 (38.6)	
	**Both**	10 (5.3)	7 (7.0)	3 (3.4)	

TB Rx, TB treatment; IQR, interquartile range. *In clinic: TB treatment initiated in study clinic; outside clinic: number of days of TB treatment received in referral clinic before TB registration and treatment continuation in the study clinic.

### Outcomes

**Figure 3:**
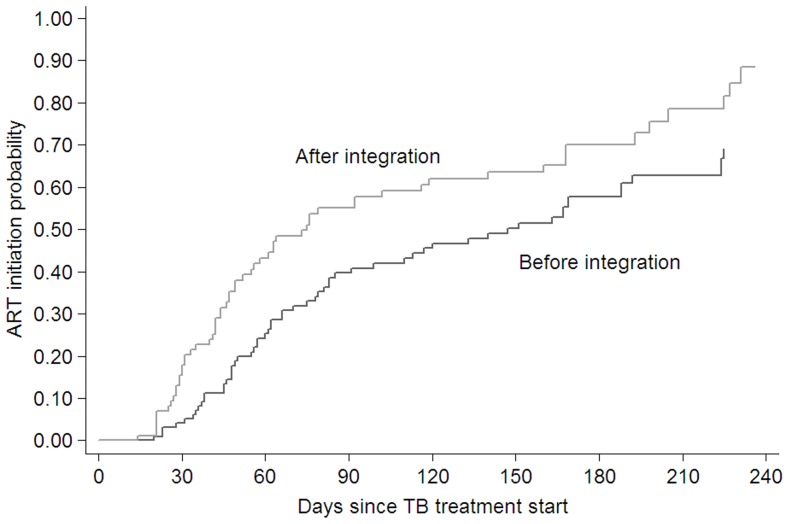
Kaplan-Meier curve for time from start of TB treatment to ART initiation for before and after service integration in Town 2 clinic from June 2008 to May 2009.

In total, 138 patients (73.4%) initiated ART. After full TB/HIV integration, the estimated median time from TB treatment start to ART initiation decreased from 147 days (95% CI 85–188) to 75 days (95% CI 52–119) **(** **Figure 3** **)**. Service integration and lower CD4 cell count were associated with shorter time to ART initiation in univariate analysis (**Table 2**). After controlling for gender, age, CD4 cell count and TB initiation, we found that patients attending the integrated TB/HIV service were 1.60 (95% CI 1.11–2.29) times more likely to start ART compared to patients attending the service before integration. In all sensitivity analyses our estimates continued to show a positive impact of the integrated service, with adjusted HRs in the range from 1.56 to 1.80 (**Table 3**).

**Table 2:** Cox proportional hazards models for the effect of TB/HIV integration and baseline covariates on time to ART.

		Univariate analysis			Multivariate analysis (n = 188)		
		cHR	95% CI	p-value	aHR	95% CI	p-value
Service integration	Before	1			**1**		**0.011**
	After	1.63	1.13–2.33	0.008	**1.60**	**1.11–2.29**	
Sex	Female	1		0.282	1		0.238
	Male	0.82	0.57–1.18		0.80	0.56–1.16	
Age; per 10 years		1.01	0.82–1.26	0.897	0.98	0.77–1.24	0.840
CD4 count; per 50 cells/µl		0.81	0.71–0.92	0.002	0.81	0.71–0.92	0.002
TB Rx initiation*; per 14 days		0.91	0.81–1.03	0.131	0.91	0.80–1.03	0.123

cHR, crude Hazard Ratio; CI, confidence interval; aHR, adjusted Hazard Ratio;

* The number of days that TB treatment was received in referral clinics before TB registration and treatment continuation in the study clinic. After adjusting for gender, age, CD4 count and previous TB initiation, patients were 60% more likely to initiate ART after service integration.

**Table 3:** Sensitivity analyses and assumptions of different models in multivariate Cox proportional hazards regression analyses.

	Adjusted HR	95% CI	p	Observations (n)
Main analysis	1.60	1.11–2.29	0.011	188
SA 1	1.64	1.13–2.37	0.009	186
SA 2	1.80	1.12–2.88	0.014	115
SA 3	1.65	1.11–2.46	0.014	157
SA 4	1.59	1.10–2.30	0.013	186
SA 5	1.56	1.09–2.25	0.016	188
SA 6	1.67	1.13–2.43	0.010	163

Adjusted hazard ratios (HR) of ART initiation after integration compared to before integration in alternative Cox proportional hazards models. The baseline model is the one presented in table 2 and includes the following variables: gender, age, CD4 count and previous TB initiation. SA, Sensitivity analysis; (n), number; **SA 1**: inclusion of the variables TB classification (pulmonary TB; extra-pulmonary TB; both pulmonary and extra-pulmonary TB) and TB patient category (new TB case; re-treatment TB case) into the model; **SA 2**: exclusion of patients transferred in from other TB services; **SA 3**: exclusion of patients with unknown exact ART initiation date during TB treatment; **SA 4**: patients with unknown exact ART initiation date during TB treatment assumed to have initiated ART in the middle of TB treatment; **SA 5**: categorization of continues variables (age, sex, TB Rx start outside of clinic); **SA 6:** only patients with CD4 cell counts ≤200 considered as according to national guidelines.

## Discussion

To our knowledge, this is the first study to assess time until ART initiation in patients before vs. after TB/HIV service integration. It highlights that TB/HIV integration is feasible in the setting described here and shows that positive outcomes for co-infected patients can be realized immediately: estimated median time from TB treatment start until ART initiation decreased from 147 to 75 days after integration. We also found that patients who received care through this integrated model were 60% more likely to initiate ART. These findings were robust to multiple sensitivity analyses.

In the fully integrated TB/HIV integration model described here, a single facility (primary health centre) and a single health care provider delivered care for both diseases. In addition to its apparent advantage in providing faster initiation of ART treatment for TB patients, this model of care has several advantages over more vertical, less integrated programs. An entire intervention package was implemented consisting of initial evaluation of clinical care and systems in the facility, followed by training and regular hands on mentoring to enhance system efficiency to cater for increasing patient load. As the number of patients on ART increased, an extra nurse and a roving part time doctor were added (after the period considered in this analysis). A recent systematic review suggests integrated models may also overcome challenges such as losing patients to follow-up during the referral process between TB and HIV services, and burdening patients with increased travel costs and more time spent in clinics [21]. In our study the proportion lost to follow-up was higher after integration, although this difference was not statistically significant. A recent study in the same setting demonstrated that assessment at an ART clinic during TB treatment reduced loss to follow-up by 80% [40]. Hermans et al. reported a decrease of TB treatment default after integration of HIV and TB services in a large urban HIV clinic in Uganda [41]. The “two diseases, one patient, one service, one appointment, one health care worker” approach also builds clinic staff’s expertise and experience in managing co-infected patients and thus better addresses patients’ considerable clinical challenges, including drug interactions and toxicity, IRIS, TB deterioration and optimal timing of ART initiation. In addition, adherence and social support interventions within integrated programs can mutually reinforce each other rather than competing for scarce resources. Lastly, integration helps improve efficiency of service delivery by avoiding duplication of logistic and administrative services.

We identified several factors that contributed to success in fully integrating our TB and HIV services: (1) getting buy-in from management, clinicians and other service providers; (2) providing training that improves competencies to treat both diseases; (3) mentoring TB staff on ARV care in the initial stages of integration; and (4) changing management processes to clarify defined areas of accountability for health staff.

Potential obstacles at the clinic level must be addressed in advance. These can include staff constraints, space, patient flow, provision of drugs, and clinical challenges. For instance, clinicians’ fear that patients with severe TB infections will develop serious ARV side effects, such as immune reconstitution inflammatory syndrome (IRIS) and drug toxic events, may lead them to delay ART initiation [28]. However, IRIS during early ART initiation is clinically manageable [42]. A study from South Africa reported that both IRIS and drug adverse events were rare in patients on an efavirenz-based treatment regimen [31]. WHO recommends ART initiation as soon as possible after TB treatment start and regardless of the patient’s immunological status [43].

Concerns that integrated clinics increase the spread of TB in immune compromised patients also need to be addressed. A study from KwaZulu-Natal found that nearly 80% of deaths in an integrated clinic were due to multi-drug and extensive drug resistant TB (MDR and XDR) [31]; however, it remains unknown whether transmission in those patients occurred at the facility level. The authors stressed that HIV patients with unknown TB infection pose the greatest risk of transmission in high HIV prevalence settings, and that integration reduces this risk through active case finding leading to earlier identification and treatment of TB. HIV-infected patients in our study were screened for TB at every visit. Moreover, diagnosed and undiagnosed HIV and TB patients also come into close contact in non-integrated clinics and in community settings (taxis, halls, homes and others), especially in high prevalence settings [44]. Thus infection control is crucial in TB and HIV high prevalence areas regardless of the level of health care integration. Whilst implementation of infection control measures is the responsibility of clinic staff, it requires a targeted focus for measures to be consistently implemented. Increased focus on infection control throughout the health sub-district of Khayelitsha was made possible thanks to the addition of an infection control officer.

### Strengths and Limitations

Randomised controlled trials are the gold standard design for evaluating complex interventions, but are not always possible because of practical, ethical, financial or methodological difficulties [45], [46] and may not accurately reflect an intervention’s feasibility or effectiveness in a real-world setting (i.e. have limited applicability) [47]. To evaluate an intervention that had already been implemented, we chose a before/after study design and used several strategies to reduce the weaknesses of this design to limit bias and confounding [46]. One strategy was to use a short (6 month) observation period before and after the intervention to limit the influence of temporal trends over results and minimize history effect bias [48]. Due to this short time span, our study was completed when national [17] and WHO [16] treatment guidelines were changed in 2010 to recommend starting ART at an earlier time (between 2 and 8 weeks after start of TB treatment) and at higher CD4 counts (<350 from <200 cells/µL before). To guard against chance findings we specified potential confounders a priori based on directed acyclic graphs [49]–[51], and demonstrated robustness to a range of sensitivity analyses. As in all observational studies, unmeasured confounding cannot be ruled out completely.

Selection bias is another potential weakness of before/after studies, since the before and after groups may differ. This study excluded about 10% of patients from the analysis because their eligibility could not be properly assessed. Most of this ambiguity arose from difficulties tracing patient records in ART referral clinics, since the previous vertical HIV and TB services each used their own unique patient identification numbers. Reasons for non-identification (and thus exclusion) included: (1) false identification information provided by patient to conceal identity; (2) deaths and LTFU occurring during the referral period; (3) patient not coming to clinic for ART services during the observation period; (4) patient accessing HIV care outside the health sub-district; or (5) inaccurate patient record keeping. Exclusion was addressed by comparing baseline characteristics of patients included and excluded. No differences were detected between included and excluded patients, nor between the before and after groups.

Most evaluation studies of TB/HIV integration to date have assessed the effectiveness of service integration using intermediate outcomes as metrics (e.g. percentage of TB patients with known HIV status); few studies have examined actual health outcomes and/or used a comparison group [21]. We used time to ART initiation as a proxy indicator for mortality and improved health outcomes. It is well documented that delayed treatment initiation for patients with CD4 cell counts below 200 cells/µl is a strong predictor of increased mortality [52], [53]. Observational studies also indicated that early ART initiation during TB treatment decreases pre-ART mortality [9], [10], [13], [42]. These findings were confirmed by a randomized controlled trial from Cambodia [42] showing that mortality of co-infected patients was reduced by 34% in the early ART initiation arm at 2 weeks versus 8 weeks, as well as by observational data [14].

Finally, this study is limited by the small sample size and the fact that it was implemented in only one site in an urban South African township. The findings need to be confirmed by larger studies in different settings.

### Conclusions

In this study TB/HIV integration resulted in immediate benefits for patients. The study further demonstrates the feasibility of integration in a peripheral primary care clinic using a nurse-led model with limited support from medical doctors. The setting is representative of many resource-constrained primary health care settings facing dual HIV and TB epidemics. Our findings add to the body of evidence supporting the rapid scale-up of service integration in settings with a high TB/HIV co-infection burden.

## Supporting Information

Figure S1 **Directed Acyclic Graph.** The following steps were used to determine the set of variables to control for confounding: (1) First, we removed all variables which satisfied both being non-ancestors of the intervention and being non-ancestors for the outcome (none variables excluded). (2) Then lines emanating from the intervention were eliminated (lines to *Time to ART initiation*, *Patient’s & health care provider’s attitude towards ART initiation*, *TB classification*) and every pair of variables with a common child or descendant was connected by undirected arcs (*Sex* and *Age*, *CD4 count at TB diagnosis* and *Patient’s & health care provider’s attitude towards ART initiation*, *TB classification* and *Patient’s & health care provider’s attitude towards ART initiation*, *TB classification* and TB treatment category, *CD4 count at TB diagnosis* and *TB treatment category*.) (3) Then all arrowheads were removed from lines. (4) Finally, variables were removed from the diagram (by deleting lines touching these variables) until the intervention and outcome was disassociated. Thus unblocked paths were closed and variables included in the final model to control for confounding were identified (*sex, age, CD4 count, TB Rx initiation*).
(PDF)

Table S1 **Comparison of activities pre- and post-integration.**
(DOCX)

Poster S1 **Tuberculosis Infection Control Policy Poster.**
(PDF)
