# Supplementary figures and images for: The Effect of Complete Integration of HIV and TB Services on Time to Initiation of Antiretroviral Therapy: A Before-After Study
Source: PLoS One. 2012 Oct 5;7(10):e46988. doi: 10.1371/journal.pone.0046988 (PMC3465310; doi:10.1371/journal.pone.0046988)

DAG 1

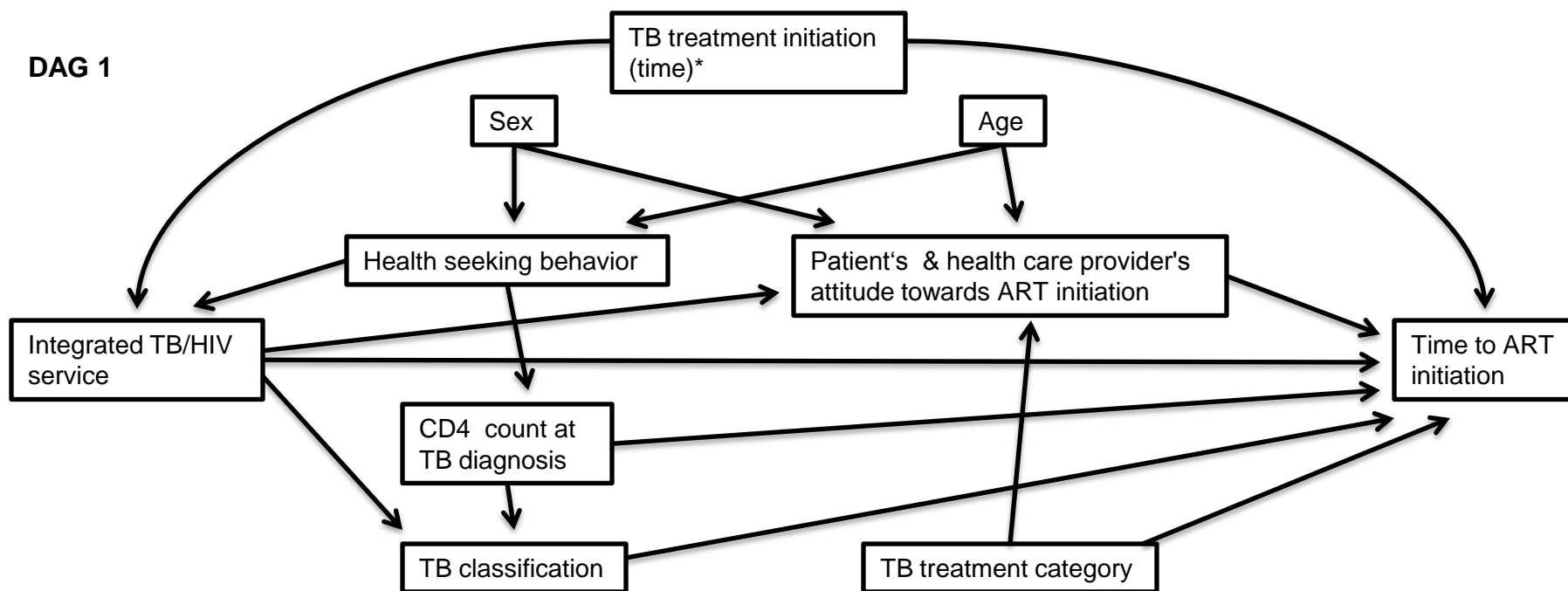

DAG 2

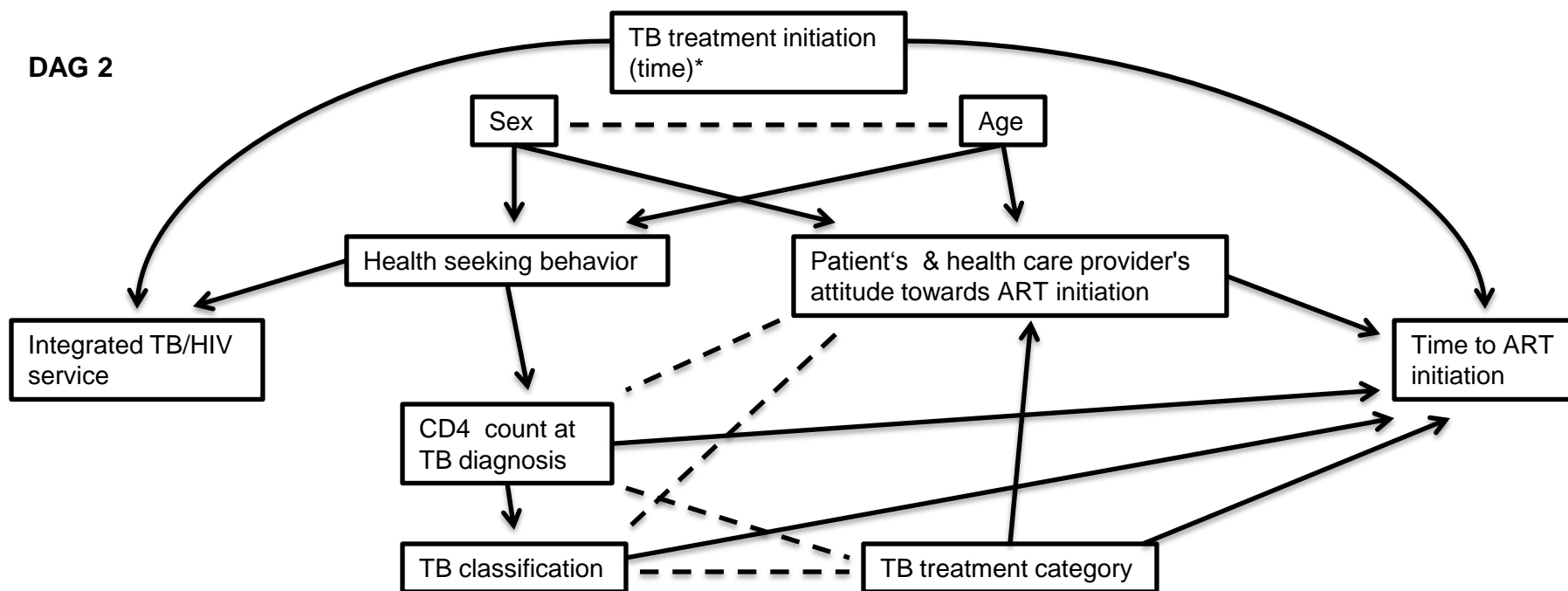

DAG 3

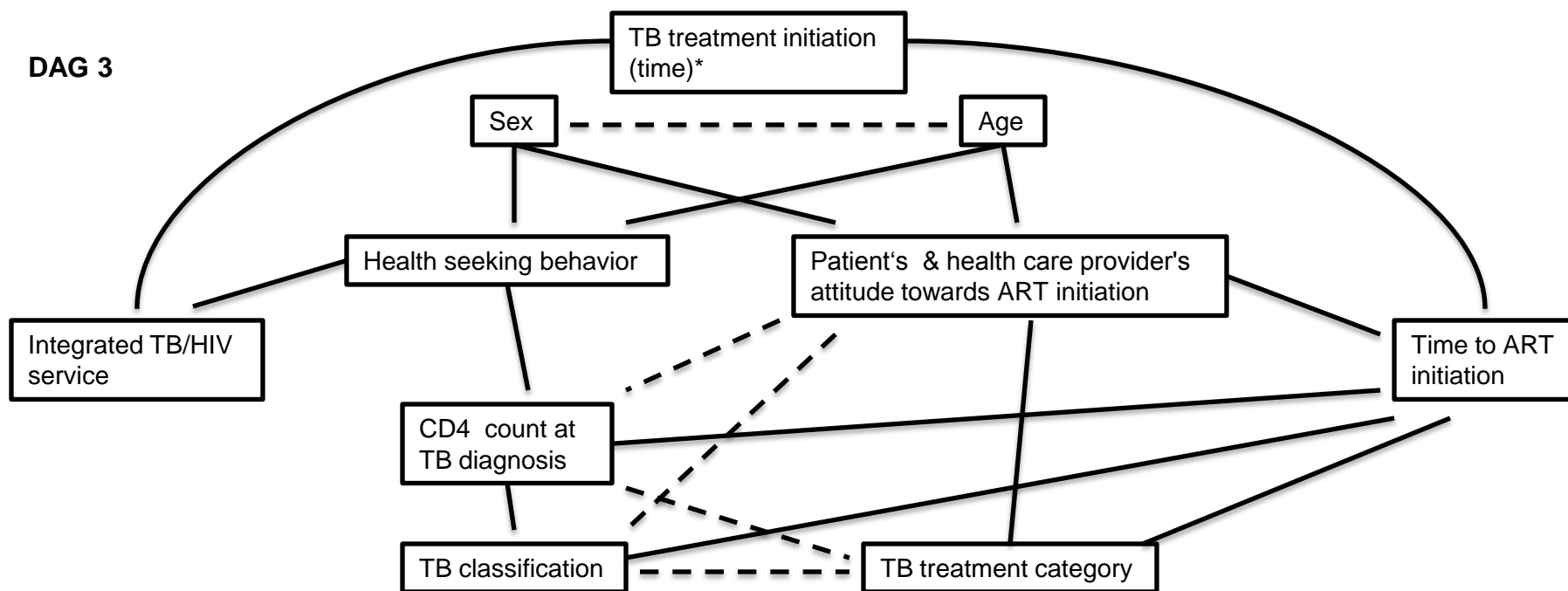

**DAG 4**

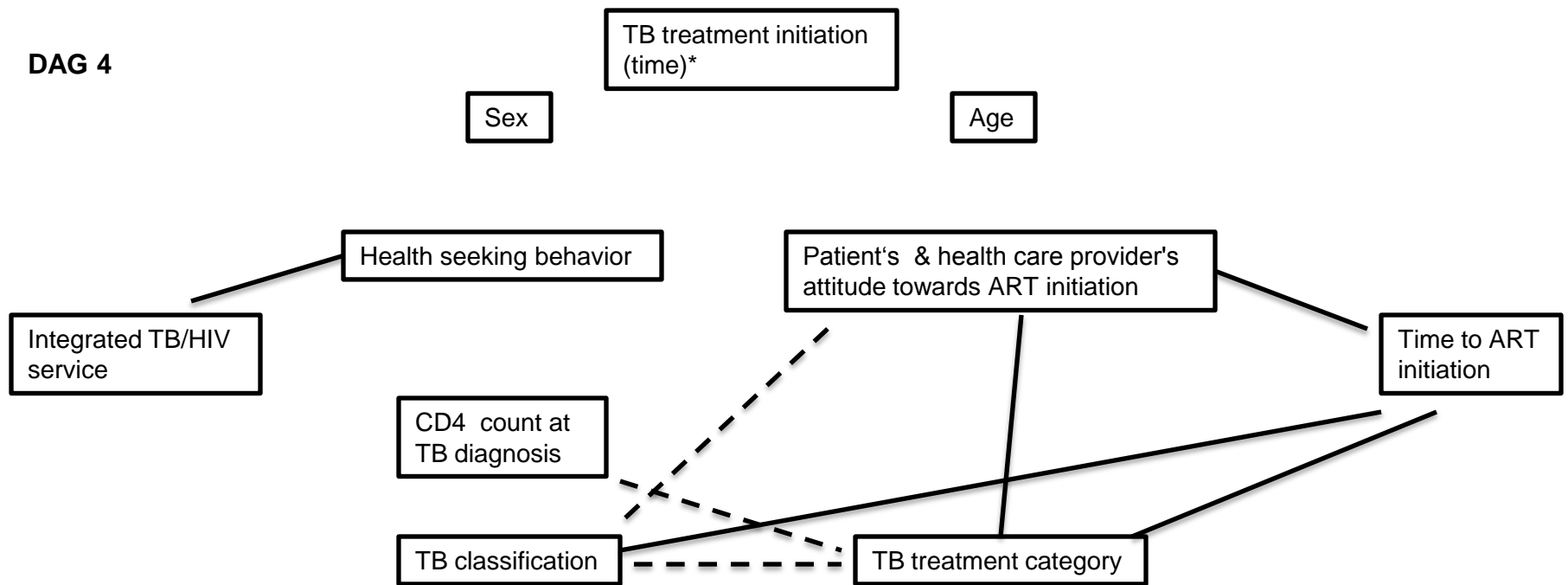

Supplement: Figure S1 — Directed Acyclic Graph. The following steps were used to determine the set of variables to control for confounding: (1) First, we removed all variables which satisfied both being non-ancestors of the intervention and being non-ancestors for the outcome (none variables excluded). (2) Then lines emanating from the intervention were eliminated (lines to Time to ART initiation, Patient’s & health care provider’s attitude towards ART initiation, TB classification) and every pair of variables with a common child or descendant was connected by undirected arcs (Sex and Age, CD4 count at TB diagnosis and Patient’s & health care provider’s attitude towards ART initiation, TB classification and Patient’s & health care provider’s attitude towards ART initiation, TB classification and TB treatment category, CD4 count at TB diagnosis and TB treatment category.) (3) Then all arrowheads were removed from lines. (4) Finally, variables were removed from the diagram (by deleting lines touching these variables) until the intervention and outcome was disassociated. Thus unblocked paths were closed and variables included in the final model to control for confounding were identified (sex, age, CD4 count, TB Rx initiation). (PDF) [file pone.0046988.s001.pdf]
