# Supplementary material for: The Effect of Complete Integration of HIV and TB Services on Time to Initiation of Antiretroviral Therapy: A Before-After Study
Source: PLoS One. 2012 Oct 5;7(10):e46988. doi: 10.1371/journal.pone.0046988 (PMC3465310; doi:10.1371/journal.pone.0046988)
Supplement: Table S1 — Comparison of activities pre- and post-integration. (DOCX) [file pone.0046988.s002.docx]

Table S1. Activities pre- and post-integration

| Activities offered pre-integration | Activities offered post-integration |
| --- | --- |
| Vertical TB care | Integrated TB/HIV care |
| Dispensing of TB drugs | Dispensing of TB drugs and ARVs |
| HIV testing and counselling | HIV testing and counselling |
| CD4 count testing | CD4 count testing |
| Cotrimoxazole prophylaxis | Cotrimoxazole prophylaxis |
| Family planning | Family planning |
| Referral to ART site | WHO clinical staging |
|  | Creatinine, haemoglobin |
|  | Adherence counselling |
|  | Initiation of ART (clinical and adherence) |
|  | 6-monthly CD4 and viral load testing |
|  | Follow-up of patients on ART (clinical and adherence) |
