# Supplementary material for: The Effect of Complete Integration of HIV and TB Services on Time to Initiation of Antiretroviral Therapy: A Before-After Study
Source: PLoS One. 2012 Oct 5;7(10):e46988. doi: 10.1371/journal.pone.0046988 (PMC3465310; doi:10.1371/journal.pone.0046988)
Supplement: Poster S1 — Tuberculosis Infection Control Policy Poster. (PDF) [file pone.0046988.s003.pdf]

Site B Ubuntu Tuberculosis Infection Control Policy

This document complies with the National, PGWC & City Health policies[1]

What is Tuberculosis (TB)?

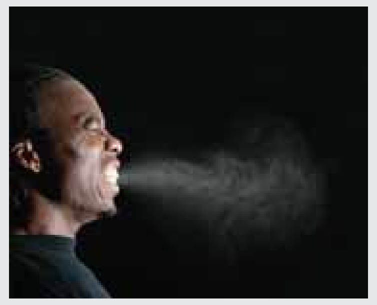

- Tuberculosis is a disease that usually attacks the lungs but can attack other parts of the body like the brain, spine, or the kidneys;
- It is a dangerous but curable disease;
- TB can be treated and cured;
- Anyone can get TB.

What is Multi-Drug Resistant (MDR) or Extremely-Drug Resistant (XDR) Tuberculosis?

- If a TB germ is drug resistant, it means that the germ can fight the medicines that we usually use to treat TB;
- A TB germ that is drug resistant is more dangerous and more difficult to treat then the "normal" TB germ;
- Multi-Drug Resistant TB and Extremely-Drug Resistant TB can be cured if treated correctly and all the medicines are taken.

How is TB transmitted? (All types of TB: Normal TB, MDR TB and XDR TB)

- TB is transmitted by air;
- When someone with TB coughs, sneezes or speaks, the TB germ is let out into the air;
- When another person breathes in these germs, TB can be transmitted.

How is TB *not* transmitted? (All types of TB: Normal TB, MDR TB and XDR TB)

- It is NOT transmitted by using utensils of someone who is sick with TB;
- It is NOT transmitted by greeting someone (shaking hands, kissing on the cheek or hugging);
- It is NOT transmitted using other peoples clothes or bed sheets;
- It is NOT transmitted by blood transfusions or by breast milk;
- TB, MDR and XDR-TB are all transmitted in the same way.

What is the risk of catching TB in Khayelitsha? This depends on two things:

- How many people have TB in Khayelitsha?
- What is being done to control the spread of TB in your home, in public places and in this facility? (see opposite)

How many people have TB in Khayelitsha?

- There are approximately 6000 cases of TB diagnosed in Khayelitsha every year;
- There were 140 cases of Multi-Drug Resistant Tuberculosis (MDR-TB) and 11 cases if Extremely Drug Resistant Tuberculosis (XDR-TB) diagnosed in Khayelitsha in 2007.

In your home and in public places, it is up to *you* to reduce the spread of Tuberculosis. You can do this by:

Being aware of the signs and symptoms of Tuberculosis, and making sure that anyone who shows any of these signs or symptoms goes to the nearest clinic for a Tuberculosis test. The earlier people get a diagnosis and treatment, the lower the chance of them transmitting the disease. The signs & symptoms of TB are:

- Cough (for more than 2 weeks)
- Night sweats
- Fever
- Weight Loss
- Tiredness or lack of energy
- Coughing up blood

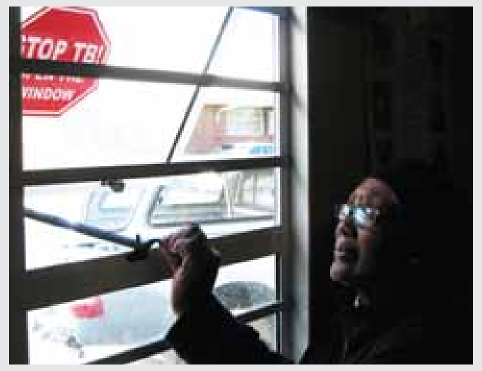

Making sure that all areas have good ventilation: Open the windows and doors to change the air so that any TB germs that are inside will flow outside;

Practising 'Cough Hygiene': People who cough should cover their mouth and nose when they do so. There are three ways of doing this:

- Use your upper arm;
- Use a tissue (throw it away afterwards);
- Use a surgical mask (throw it away afterwards);

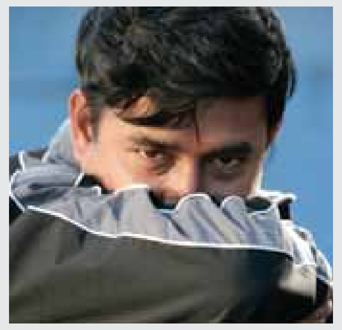

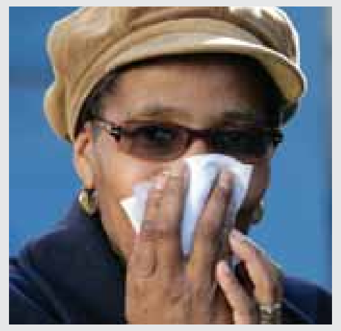

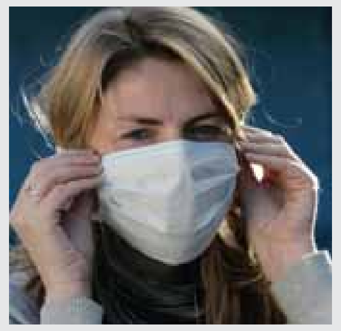

What is being done to reduce the risk of transmission of TB in this facility?

What is Infection Control (IC)?

Infection Control is the prevention of transmission of infectious diseases, such as Tuberculosis, Influenza, HIV, diarrhoeal diseases, etc. This poster is about just one of these infectious diseases: Tuberculosis.

Who is responsible for Infection Control? – Everyone!

In this facility, the Infection Control Committee consists of the Facility manager, the Health & Safety Representatives, the Cough Officer, the TB Nurses and a representative from the local community;

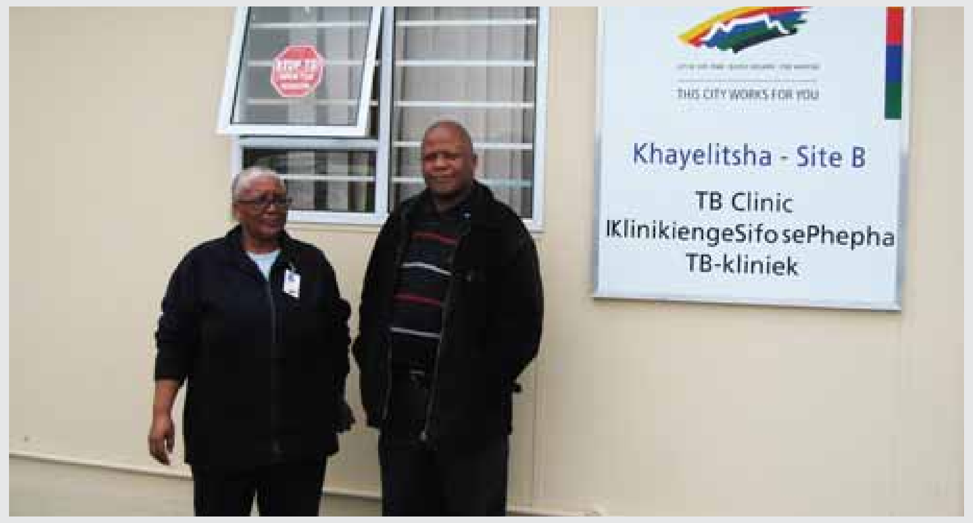

There are three different types of infection 'controls' in place in this facility:

The staff are expected to adhere to all of these controls:

- Administrative Controls
- Environmental Controls
- Personal Respiratory Protection

Administrative Controls – these are the most important!

Control	Description of Control	How to do this?	Who will do this?
Perform a TB IC Risk Assessment every 6 months	Perform a formal risk assessment of the facility every 6 months in order to: identify the TB burden, the high risk and low risk areas, the patient flow etc. essentially all the elements that define the risk of nosocomial transmission of TB[2]	Use the 'Facility Risk Assessment Tool for Tuberculosis' (FRATT). A questionnaire specially designed for this purpose	Facility Manager & Program Manager
Monitor prevalence of TB & DR TB	Case reporting of TB and DR-TB cases seen and treated in the clinic and analysis of the results	Fill in the DR-TB register	TB Staff
Monitor TB IC standard of facility	Ensure that IC measures that are in place are correctly followed, identify & document potential problems, be reactive to changing prevalence etc.	Use this poster! Hold monthly meetings of the Infection Control Committee	Infection Control Committee
Screen Health Care Workers (HCW) for TB	Ensure that HCW are aware of signs & symptoms of TB so they can be proactive about getting themselves tested	In addition to being proactive about self diagnosis, all HCW should fill in a questionnaire every 6 months	Facility Manager
Ensure HCW sickness cover	All staff should be aware that if they catch TB then they have a right to receive treatment and workers compensation allowance	The latest version of 'Compensation for Occupational Injuries & Diseases Act 1993' should be available in the facility for staff to read	Facility Manager
Provide HCW education	There should be appropriate in-service training for all HCW on TB and other infectious diseases	In-service training should be organised four times per year for all staff	Facility Manager
Provide patient education	Patients should be educated about TB and other infectious diseases and ways to prevent them, e.g. Cough Hygiene	An educator should do this every morning for one hour in each waiting room	The facility manager can delegate this to a local NGO
Provide community education	The local community should be educated about infectious diseases and ways to prevent them, e.g. Cough Hygiene	The Facility should take the initiative to go into the community and carry out talks, meetings etc.	The Infection Control Committee
Assign Responsibility for Infection Control	Each facility should have an Infection Control Committee which should meet monthly to discuss Infection Control issues	The committee should be made up of: The Facility Manager, the Health & Safety Reps., the TB Nurses and representatives from the local community	Facility manager
Screen<br>Educate<br>Separate	Appoint a 'Cough Officer' to screen patients who arrive in the main waiting area for signs of TB, educate them on cough hygiene and separate / fast track them	Elect a Cough Officer from among the staff and provide them with the necessary training	The facility manager can delegate this to a local NGO
Cohort Patients – Separate them into different groups	Cohort patients into different groups according to the risk they represent and allocate them appointments at different times of the day to avoid mixing of the populations	All DR TB patients should come at the end of the morning, when the facility is less crowded. Children should not come on the same day as DR TB patients	TB Sister, TB Dr.
Organise patient flow	Study patient flow and re-organise it so as to avoid TB patients mixing with non-TB patients	Move services to different rooms where necessary	Facility Manager & Cough Officer
Diagnose TB patients rapidly	Ensure that all the clinical staff are aware of the signs and symptoms of TB and diagnose rapidly	Specialist education of all HCW in diagnosing TB	TB Doctor
Confirm diagnosis rapidly	Ensure that all sputum smear & culture samples are rapidly sent to the lab and analysed. The results should be transmitted to the clinician within 3 working days for sputum results	Ensure that all staff are aware of the procedure, that correct sample pots and forms are available. Ensure the lab performs correctly	TB Sister, Facility Manger & TB Doctor
Follow-up contact cases of patients sick with TB	Ensure that all contacts of newly diagnosed TB patients are screened for TB[3]	Allocate responsibility for this, and a tool – the 'Contact Case Follow-up sheet'	TB Sister, TB Adherence Counsellor
Use a protocol for sputum induction	Sputum induction is a high risk procedure and should be done outside under HCW supervision	Install external sputum booths which can be seen from the sputum room	Facility Manager
Display TB Signage & Literature	Display TB signage & literature for both patients and HCW to read for example "TB in Our Lives" by Treatment Action Campaign	Obtain existing literature from City, Govt. and NGOs	Facility Manager
Enforce a maintenance policy for environmental controls	Establish a maintenance plan for the environmental controls (ventilation/ fans etc.) in the facility and follow up that it is carried out	Electrical fans should be checked every six months by a technician	Facility Manager
Ensure that there is a budget for IC	Establish a budget specifically for IC, to include allowance for rehabilitations, training, literature, respirators and masks etc.	Calculate the needs and the cost of these needs. Transmit this to management	Facility Manager

Environmental Controls[4]

Control	Description of Control	How to do this?	Who will do this?
Maximise natural ventilation	Open the existing windows and doors as much as possible in order to maximise natural ventilation.	Put up 'Stop TB – Open a window' stickers in the facility and ensure that HCW know why this is important	Facility Manager
Avoid being downwind from a TB patient	Arrange the room in such a way that the air flow goes from the HCW to the patient	Establish the direction of air flow with an incense stick and move your desk/table accordingly	All Staff

Personal respiratory protection

Control	Description of Control	How to do this?	Who will do this?
Patients to wear surgical masks	Confirmed and suspect TB patients should be educated in the use of and given surgical masks to wear when they are inside the clinic	Install IC cabinet displaying poster of Cough Hygiene and dispensing surgical masks (& tissues, TB literature etc.)	Cough Officer
Staff to wear N95 respirators[5] 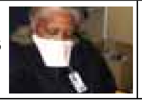	HCW to be fit tested, educated in the use of, and provided with N95 respirators, which they should wear when in contact with suspect or confirmed TB patients	Ensure a supply of N95 respirators and arrange for a fit test program	Facility manager

1 National Infection Prevention and Control Guidelines for TB, MDRTB and XDRTB, June 2007. Guidelines for the prevention of transmission of TB in Health Care Facilities in the Western Cape Province. CT 07/01 Guidelines for the prevention of transmission of Tuberculosis in Clinics. CT 04/79 Infection prevention and control policy

2 'Nosocomial' means transmission of a disease in a healthcare facility.

3 A 'Contact' is a person who lives with someone who is sick with TB

4 Notes on environmental controls:
UVGI lighting is not considered appropriate for this context because:
- Risk of Photokeratitus, Erythema & Skin carcinoma in patients and HCW if not installed properly;
- Only effective if air is pushed into and stays in the 'kill zone' for a certain amount of time (with extraction fans it becomes less effective);
- Needs to be maintained regularly – wipe lamps with alcohol once per month;

High Efficiency Particulate (HEPA) filters, Electro Static Precipitators (ESP), Negative Ionizers, Ozone generators, UVGI light boxes etc. are not considered appropriate for this context because of their high purchase and maintenance cost, and unproven effectiveness.

5 The PGWC has elected to use the regular size N95 respirators manufactured by Kimberly Clerk. Based on the experience of the IC practitioner, 90% of HCW pass the fit test with this mask. However, a solution needs to found for the remaining 10% (e.g. 'Small' N95 respirators by Kimberly Clerk – currently not available in SA, or respirators by 3M)

If you have any more questions, please do not hesitate to contact the MSF Infection Control Practitioner:

Tel: 021 364 5490 (office)
or 0784 908 102 (mobile)
Email: msfb-khayelitsha-ic@msf.org.za

MEDECINS SANS FRONTIERES
